# Comparative analysis of transcriptomic profile, histology, and *IDH* mutation for classification of gliomas

**DOI:** 10.1038/s41598-020-77777-6

**Published:** 2020-11-26

**Authors:** Paul M. H. Tran, Lynn K. H. Tran, John Nechtman, Bruno dos Santos, Sharad Purohit, Khaled Bin Satter, Boying Dun, Ravindra Kolhe, Suash Sharma, Roni Bollag, Jin-Xiong She

**Affiliations:** 1 Center for Biotechnology and Genomic Medicine, Augusta, USA; 2 Jinfiniti Precision Medicine, Inc., Augusta, USA; 3 Department of Obstetrics and Gynecology, Medical College of Georgia, Augusta, USA; 4 Department of Undergraduate Health Professionals, College of Allied Health Sciences, Augusta, USA; 5 Department of Pathology, Medical College of Georgia, Augusta University, 1120 15th Street, Augusta, GA 30912 USA

**Keywords:** RNA sequencing, Cancer genomics

## Abstract

Gliomas are currently classified through integration of histology and mutation information, with new developments in DNA methylation classification. However, discrepancies exist amongst the major classification methods. This study sought to compare transcriptome-based classification to the established methods. RNAseq and microarray data were obtained for 1032 gliomas from the TCGA and 395 gliomas from REMBRANDT. Data were analyzed using unsupervised and supervised learning and other statistical methods. Global transcriptomic profiles defined four transcriptomic glioma subgroups with 91.4% concordance with the WHO-defined mutation subtypes. Using these subgroups, 168 genes were selected for the development of 1000 linear support vector classifiers (LSVC). Based on plurality voting of 1000 LSVC, the final ensemble classifier confidently classified all but 17 TCGA gliomas to one of the four transcriptomic profile (TP) groups. The classifier was validated using a gene expression microarray dataset. TP1 cases include IDHwt, glioblastoma high immune infiltration and cellular proliferation and poor survival prognosis. TP2a is characterized as IDHmut-codel, oligodendrogliomas with high tumor purity. TP2b tissue is mostly composed of neurons and few infiltrating malignant cells. TP3 exhibit increased NOTCH signaling, are astrocytoma and IDHmut-non-codel. TP groups are highly concordant with both WHO integrated histology and mutation classification as well as methylation-based classification of gliomas. Transcriptomic profiling provides a robust and objective method to classify gliomas with high agreement to the current WHO guidelines and may provide additional survival prediction to the current methods.

## Introduction

Gliomas are neoplasms that mostly arise from the cerebral hemisphere of adults^1^. They exhibit highly variable response to chemoradiation therapy and survival prognosis^2–4^. It is important to accurately classify these tumors for better treatment and prognostication. Gliomas are classified using a combination of histology and molecular testing^1,5^. Histologic diagnoses include glioblastoma, astrocytoma, and oligodendroglioma^6^. However, inter-observer disagreement with histologic diagnosis is quite high^7^. Glioma classification became more automated and systematic^8,9^ with the discovery of mutations in the isocitrate dehydrogenase (*IDH*) gene^10^ (IDH mutated, IDHmut)^5^ of non-gliobastomas, as compared to IDH wild type (IDHwt) glioblastomas^11–13^ and co-deletion of chromosome arms 1p/19q in oligodendroglioma^14–16^. IDHwt glioblastomas are additionally evaluated for *O*(6)-methylguanine-DNA methyltransferase (MGMT) promoter methylation to determine likelihood of tumor response to the alkylating agent temozolomide^17,18^.

Yet, mutational classifications are suboptimal prognostic markers. Glioblastoma patients with IDHwt are among the worst prognosis gliomas; however, many good prognosis gliomas (i.e. grade 1 pilocytic astrocytomas and gangliogliomas) are IDHwt^19^ and a subgroup within IDHwt glioblastomas has been described as pilocytic astrocytoma-like, with good survival prognosis^9^. WHO has identified the need of additional molecular markers to delineate the poor prognosis IDHwt patients^19^. Furthermore, secondary glioblastomas occasionally arise from a primary astrocytoma lesion^20^. These carry the poor prognosis of glioblastomas, but are IDHmut^8^.

Current glioma diagnosis using histology and IDH-codeletion testing is sufficient for most cases, but there are inconsistencies. Histologic diagnoses depend on human pattern recognition and is prone to errors^21,22^ and biases^23,24^, which are especially apparent under time constraints^25^. It is therefore necessary to develop objective and automated measures of tumor classification. Much progress has been made to use machine learning to automate and objectify cancer diagnosis and subtyping^26–28^. In 2010, Verhaak et al.^29^ described the transcriptomic classification of glioblastomas into four clusters using consensus clustering^30^. However, since this method was developed from glioblastomas, it may provide limited use in classifying other gliomas. Additionally, some reports have described limitations and potential inaccuracies in using the consensus clustering algorithm^31^. Recently, two reports have highlighted the potential use of DNA methylation to classify central nervous system (CNS) tumors with high accuracy and then to identify new subclasses^9,32^. Both reports showed overall agreement with IDH-codeletion classes, but the minor disagreements and new subclasses are associated with changes in grade or prognosis. The TCGA reported six glioma DNA methylation classes. Three of the clusters mainly subclassify glioblastomas. Interestingly, even though patients with IDH mutated gliomas usually have better survival prognosis than patients with IDH wt gliomas, TCGA reported the LGm1 DNA methylation cluster identifies IDH mutated cases associated with worse survival prognosis^9^.

While these molecular classifications provide an important step forward for automated and objective glioma classification, a diagnosis from one “-omic” platform is not enough for defining a tumor class. Logically, with each -omic platform which agrees on the classification of sample “x” in class “y”, the confidence in sample “x” truly belonging to class “y” increases. Hence, defining distinct tumor classes requires multiple -omics platforms to corroborate the same classification. Confident tumor classification from multiple -omics platforms provides two potential benefits to cancer research. First, the identification of molecular classes may highlight new targetable pathways for therapeutic intervention^33^. Second, accurate tumor classification is necessary to perform many statistical analyses involving these tumor types. Differential frequencies and expressivities innate in tumor subtypes may confound statistical association analyses. For example, if a cancer type has an un-identified poor prognosis molecular subtype, a project to determine genes associated with poor prognosis in a cancer may only identify genes enriched in this molecular subtype. This is well studied in terms of population stratification for genome wide association studies (GWAS)^34^.

In order to better define molecular subtypes of gliomas, we sought to develop a transcriptome-based glioma classification method using unsupervised classification techniques and compared the findings to other glioma classification methods. While glioblastomas have been characterized using transcriptome subtypes^29,35^, this has not been performed comprehensively for all gliomas.

## Methods

### TCGA dataset

TCGA Glioma gene expression data, which contains both RNAseq and gene expression microarray data combined through Empirical Bayes, was downloaded from Ceccarelli et al. 2016^9^. The final dataset contained 1032 samples and 12,717 genes. TCGA Glioma data was centered and scaled. Clinical data were downloaded from the same source and matched to the processed TCGA Glioma data.

### REMBRANDT dataset^36^

Normalized gene expression data from fresh frozen tumor were downloaded from E-MTAB-3073 through ArrayExpress and clinical data were downloaded from G-DOC^37^ (https://gdoc.georgetown.edu/gdoc/), both on September 19, 2018. The expression data were normalized using Expression Atlas, which applied RMA from “oligo”^38^ (v 1.36.1). Outlier Detection was performed with boxplots (Kolmogorov–Smirnov statistic K_a_), distances between arrays (35 detected), and MA plots (Hoeffding’s statistic D_a_ > 0.15)^8^ on Expression Atlas. In total, 43 outlier samples were removed. Three hundred and ninety five cases overlapped between the outlier removed expression data and clinical data. For genes with multiple probes, the probes with highest mean intensity was used and all others removed. REMBRANDT data was standardized before supervised classification. Only 161 of the 168 genes used for supervised classification were present in the microarray dataset.

### Density-based UMAP (DBU) algorithm

The optimal parameters for uniform manifold approximation and projection (UMAP) implemented in the “umap”^39^ package were determined using a grid search. The grid search parameter optimization was performed by visual inspection to maximize cluster density and intercluster distances on UMAP plots. Parameters were number of nearest neighbors (n_neighbors), minimum distance (min_dist), number of genes, and distance metric (metric). The final selected parameters were 1000 random genes, 5 nearest neighbors, 0 minimum distance, and the “manhattan” metric.

The two dimensional data from UMAP was entered into the DBSCAN algorithm from the “fpc” package^40^. The minimum points parameter was empirically determined as 100 based on the approximate number of cases in each of the three groups and the optimal eps parameter was 1.55 based on the elbow of the k nearest neighbors distance plot. The density-based UMAP (DBU) algorithm combines the UMAP and DBSCAN steps repeated 1000 times based on subsampling of 1000 random genes. Hierarchical clustering of the DBU iterations revealed a subset of the iterations required manual switching of group assignment to agree with the majority of iterations. Thirty-four models that only identified two groups were removed from subsequent analyses.

The samples were classified based on plurality voting, where the sample class is assigned as the class with the most votes from the DBU iterations. Samples are designated as “ambiguous” if less than 70% of the models assign the sample to one specific group or if the most commonly assigned group was Group 0 (ambiguous on the DBC algorithm). Most (96.6%) iterations/models classified the vast majority of patients into three main groups with a small number of patients that cannot be unambiguously assigned to one of the three groups. Some UMAP models showed potentially further heterogeneity within Group 2. Further analysis identified relevant molecular differences for two clusters within Group 2. Thus, the final groups were designated TP1, TP2a, TP2b, and TP3 to delineate the two subgroups of group 2.

### Ensemble transcriptomic classification (ETC) algorithm

One hundred and 68 genes were identified for use in supervised classification using two complementary methods. First, significantly differentially expressed genes amongst the four DBU groups were identified through LIMMA analysis^41^ and 26 genes were manually selected based on relevance to brain cancer. The remaining 142 genes were selected using recursive feature elimination with a support vector classifier. These genes were divided into six groups based on the expression differences among the subtypes. For each supervised model, half of the genes in each of six gene groups were randomly selected and then recursive feature elimination was applied removing five genes per iteration until optimal accuracy is reached with the minimal number of genes using “sci-kitlearn^42^”. This was repeated 1000 times, resulting in a dictionary with 1000 entries each with between 29 and 79 genes of the 168 genes. One thousand linear support vector classifiers (LSVC) were developed from the dictionary and the mean accuracy from threefold cross validation was used to remove any models with average accuracy less than 95%. All 1000 models passed this step and average model accuracy was 97.6%.

In order to decrease the potential of overfitting, data were split into fourfolds, where three folds were trained on the unsupervised model classes and the supervised models predicted on the remaining fold. In this way, no sample was used for both training and making classification calls. This results in calls for each sample from 1000 linear SVC models. Models were combined into one ensemble model using a plurality voting method which reports the most popular class and the proportion of the 1000 LSVC models which agree on this most popular class. A confidence score is calculated by taking the proportion of models classifying samples into the most popular class divided by the proportion of models classifying samples into the second most popular class. If the confidence score is greater than 3, then the ensemble model classifies the sample into the most popular class. If the confidence score is less than or equal to 3, than the ensemble model prediction is “ambiguous”.

### Mutation analysis

Mutation data for TCGA Glioma data was downloaded from UCSC Xena^43^ (https://xenabrowser.net/datapages/) GBMLGG somatic non-silent mutation (gene-level) PANCAN AWG TCGA Hub for 461 cases. “0” is “no mutation” and “1” is “mutated.” Genes with no mutation in any samples were removed. Chi-squared analysis for one vs rest was performed and p-values were adjusted with Benjamini-Hochberg^44^.

### Pathway analysis

LIMMA^41^ was used for differential expression analysis comparing each group vs the rest (i.e. TP1 vs TP2a, TP2b, and TP3) after removal of ambiguous samples based on the supervised classification.

Results from LIMMA were used for GSEA^45^ pathway analysis. Gene Set Enrichment Analysis (GSEA) was implemented with the “fgsea”^46^ R package. Reactome^47^ pathways were downloaded from MSigDB (674 pathways). Pathways were filtered for minimum size of 15 genes and maximum size of 500 genes, resulting in 440 pathways. One hundred and ninety-three pathways were enriched in at least one group. There were 123 significantly enriched pathways in TP1, 57 significantly enriched pathways in TP2a, 146 significantly enriched pathways in TP2b, and 26 significantly enriched pathways in TP3. Significant pathways are those with Benjamini-Hochberg-adjusted p-values < 0.05.

### Survival analysis

We modeled survival with Kaplan–Meier (KM) and Cox proportional hazards and tested for significance with the log rank test all using the “survival” R package^48^. KM plots were made through the “survminer” package^49^.

### Tumor purity

Tumor purity measures were obtained from TCGA Ceccarelli et al. data based on the ABSOLUTE algorithm^50^, which uses DNA copy number data to calculate tumor purity, and the ESTIMATE algorithm^51^, which uses DNA methylation data to calculate the proportion of immune and stromal cells in the bulk tumor.

All statistical analyses were performed using the R language and environment for statistical computing (R version 3.5.1; R Foundation for Statistical Computing; https://ww.r-project.org)^52^.

## Results

### Unbiased transcriptomic classification of gliomas

**Figure 1:**
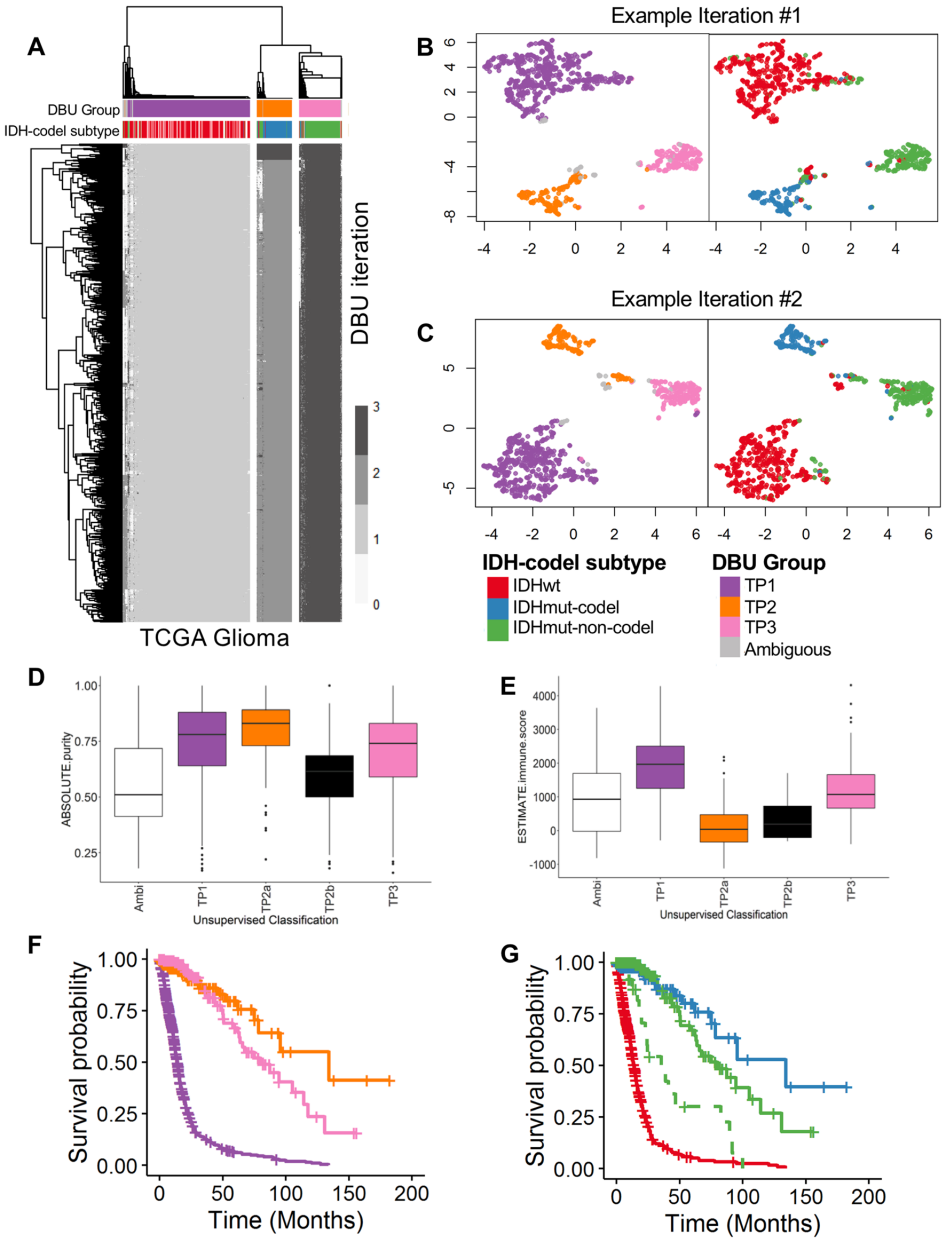
Unsupervised dimension reduction and density-based consensus clustering of TCGA Gliomas. (**A**) Heatmap showing clustering results from 1000 models of 1000 randomly selected genes. Rows represent individual model iterations and columns represent TCGA cases. Cluster assignment for each case and by each model is represented by different shades of grey. Samples are designated as “ambiguous” if less than 70% of the models assign the sample to one specific group. The top 34 models only identified two main groups and were removed from consensus calling. Consensus calls are shown on the top bar and are compared to IDH mutation and 1p/19q codeletion status. (**B**) One representative DBU iteration. The left panel shows unsupervised TP clusters and the right panel shows the same clustering with samples color-coded for IDH-codel status. (**C**) A second representative DBU iteration organized similarly to (**B)**, showing four possible clusters. (**D**) Tumor purity using ABSOLUTE measure (downloaded from TCGA) based on unsupervised transcriptome classification. Anova (AOV) p = 2.7e − 14. The whole cohort tumor purity was 0.77 [0.62–0.87] (median [IQR]), while the Ambiguous group tumor purity was 0.51 [0.41–0.72] and the TP2b group tumor purity was 0.62 [0.50–0.69]. (**E**) Tumor immune infiltration using ESTIMATE measure (downloaded from TCGA) based on unsupervised transcriptome classification. AOV p < 2e − 16. (**F**) Kaplan–Meier (KM) survival estimates of overall survival for our transcriptomic classification, TP1 (purple, univariate HR 11.2 [7.0–17.8]), TP2 (pink, ref), TP3 (orange, 1.4 [0.8–2.5]). (**G**) KM curves of overall survival highlighting the 27 IDHmut-non-codel patients who are classified as TP1 (dashed green line). These cases have worse survival prognosis than other IDHmut cases (median survival time = 35.4 months vs 79.9 months) and survival is more similar to IDHwt cases (13.8 months). IDHwt TP1 (red), IDHmut-non-codel TP3 (solid green), and IDHmut-codel TP2 (blue). Ticks represent censored cases.

We identified tumor subgroups of the TCGA Glioma gene expression data, using 12,717 genes and an unsupervised classification algorithm combining density-based clustering (DBC)^40^ and uniform manifold approximation and projection (UMAP)^39^ (termed “DBU”). Our pipeline randomly samples 1,000 out of 12,717 genes and applies the UMAP dimension reduction algorithm, followed by a DBC algorithm, for 1000 iterations. Most (96.6%) iterations/models classified the vast majority of patients into three main groups with a small number of patients that cannot be unambiguously assigned to one of the three groups (Fig. 1A). UMAP for one representative model, which shows three main groups, is shown in Fig. 1B, while another representative model shows potentially further heterogeneity within Group 2 (Fig. 1C). The 34 remaining models only identified two major groups and were removed from further considerations.

The final DBU group assignment is determined by plurality voting, where the “winner” needs support from 70% or more of the 966 models. If no group wins at least 70%, the patient is called “ambiguous”. Plurality voting assigned 603 (58.4%) patients to “Transcriptome Profile 1” (TP1), 174 (17.0%) patients to “TP2”, 208 (20.1%) patients to “TP3” and 47 (4.6%) samples as “ambiguous.” The ambiguous samples may have been more difficult to classify because they had lower tumor purity than other samples that is not related to immune infiltration (Fig. 1D,E).

### High concordance between transcriptomic and WHO classifications

**Figure 2:**
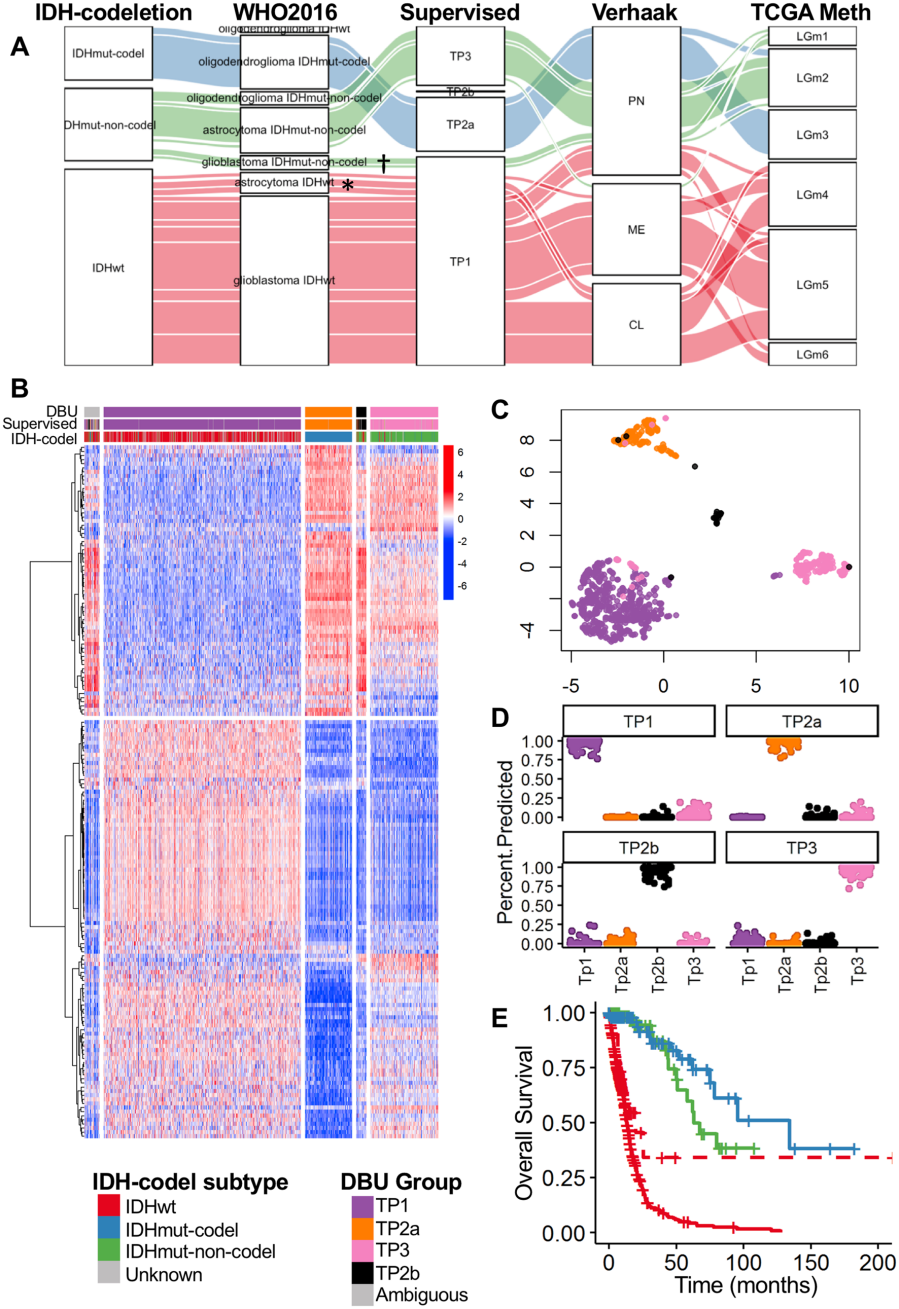
Gene selection and supervised model classifications on TCGA Glioma dataset. (**A**) Alluvial diagram showing the consistencies and discrepancies between different classifications methods for TCGA Glioma dataset. Colors represent the IDH-codeletion status of the samples. *indicates the IDHwt Astrocytoma cases †indicates the IDHmut Glioblastoma cases. (**B**) Expression heatmap of TCGA Glioma cases (columns) for 168 selected genes (rows). Sample categories are shown on the top bar. (**C**) One representative UMAP for TCGA Glioma samples based on supervised model. Colors represent the unsupervised consensus classifications. (**D**) Scatter plots showing the percent of the 1000 models that cluster each sample (one dot) as belonging to each group. Samples classified as ambiguous by the supervised consensus are not shown here. (**E**) Kaplan–Meier survival estimate of TCGA overall survival data comparing IDHwt astrocytoma cases (dashed red), median survival = 19.9 months) to IDHmut-non-codel astrocytoma (green, median survival = 62.9 months) (LRT p = 2e-05). IDHwt glioblastoma (red) and IDHmut-codel oligodendroglioma (blue) are also shown as reference.

The 2016 WHO Classification of Tumors of the Central Nervous System^5^ recommends using IDH1/2 mutation and 1p/19q co-deletion (codel) status in combination with histology to classify infiltrating gliomas. Our transcriptomic classification is 91.4% concordant with IDH-codel classification (Fig. 2A, Supplementary Table 1). 90.9% of IDHwt patients are classified as TP1, 92.3% of IDHmut-codel patients are classified as TP2, and 75% IDHmut-non-codel patients are classified as TP3. Consistent with their IDHwt status, the TP1 patients also have the worst survival prognosis (Fig. 1F).

Some important discrepancies were noted. First, 27 of 252 (10.7%) IDHmut-non-codel patients were classified as TP1. As shown in Fig. 1G, they have survival similar to IDHwt TP1 patients, but worse survival than IDHmut-non-codel patients, suggesting that our transcriptomic classification reflects better glioma patient survival prognosis than the IDH-codeletion-based classification.

The second major discrepancy between the transcriptomic and IDH-codeletion-based classification systems involves 31 patients (23 IDHmut-non-codel and 8 IDHwt) that were classified in the TP2 group, which is predominantly IDHmut-codel (144 of 175 or 82.3%). These 31 patients are within a distinct subgroup on a subset of UMAP models (Fig. 1C). Thus, we designated this subgroup as TP2b which contains patients with IDHwt or IDHmut-non-codel. The remaining 144 TP2 patients all have IDHmut-codel status and will be referred to as TP2a. TP2b samples have significantly lower tumor purity, not associated with increased immune or stromal infiltration (Fig. 1D,E).

### Supervised classification with 168 genes

Although the unsupervised models are powerful and unbiased tools to assess the global transcriptomic structures within gliomas, the approach is not ideal for classifying future tumors owing to the potential of batch variability affecting classification and the need to re-run the full algorithm to classify each new sample. Therefore, we employed a combination of differential expression analysis, forward feature selection, recursive feature elimination and manual curation to select 168 genes that were used to develop supervised classifiers (Fig. 2B, Supplementary Table 1, 2). UMAP of all 168 genes recapitulates the four transcriptomic groups defined by the unsupervised classifier (Fig. 2C).

To develop a generalizable classifier, we generated 1000 linear support vector classifiers (LSVC), each 38 genes on average from the 168 gene set (Supplementary Table 3), trained using all patients after removing the 47 ambiguous patients based on the DBU iterations. The 168-gene supervised ensemble model based on plurality voting is henceforth referred to as ensemble transcriptomic classification (ETC).

ETC had an agreement of 99.4% when compared to the unsupervised transcriptomic classification. All patients in TP1, TP2a and TP3 groups were unambiguously assigned to their respective groups (Fig. 2D). However, 5/31 patients (16.1%) in TP2b could not be unambiguously assigned to TP2b. ETC also confidently assigned 30 of the 47 cases that cannot be unambiguously assigned by the unsupervised DBU models, leaving a total of 17 patients that cannot be confidently assigned to a group. ETC is used for subsequent analyses. The unsupervised and supervised classifications, histology, and IDH-codeletion status for all TCGA samples are provided in Supplementary Table 4.

### ETC groups resolve discrepancies between histological and mutation-based classifications

WHO-defined mutation and histology classifications for the TCGA samples have an 82.0% concordance rate, which is lower than the concordance rate between ETC and histology or mutation, which are 90.2% and 95.5%, respectively (Supplementary Table 5).

ETC also resolves several histology-mutation discrepancies. The 27 IDHmut-non-codel cases classified as TP1 and, with poor survival similar to IDHwt TP1 cases (Fig. 1G), are histologically diagnosed as glioblastoma. These IDHmut glioblastomas are considered “secondary glioblastomas” arising from lesions originally classified as astrocytomas. ETC agrees with the histological diagnosis and the survival prognosis for these patients. There are 44 cases histologically diagnosed as astrocytoma but with IDHwt (Fig. 2A). This is contradictory because IDHwt cases are typically glioblastoma, which has worse survival than astrocytoma. Of these cases, 31 are classified as TP1, 2 in TP3, 5 in TP2b, and 6 are ambiguous. Thus, in the TCGA dataset, ETC mostly agrees with IDHwt status and would predict the worst prognosis, which is true for these patients (Fig. 2E). In both discrepancies, ETC agrees with the classification that correctly predicts survival prognosis, suggesting that ETC is complimentary to both histologic and mutation-based classifications.

The remaining disagreements between histology and mutation status are more difficult to resolve through survival analysis since they are between astrocytoma and oligodendroglioma cases. The WHO recommends following the IDH-codeletion status to diagnose these cases and, indeed, ETC agrees with IDH-codeletion status as well.

### ETC groups are distinct from previously reported transcriptomic clusters

We compared our ETC classifications to the three transcriptomic clusters previously reported by Verhaak et al., “Classical”, “Mesenchymal”, and “Proneural”^29,35^. We refer to these four groups as the “Verhaak” classification. In the TCGA cohort, the concordance between our two methods was 64% while the concordance between the IDH-codel and the Verhaak classification was 65% (Fig. 2A, Supplementary Table 5). Nearly all Classical (186/186) and Mesenchymal (190/205) cases were classified as TP1. Proneual had even contributions from TP1, TP2a, and TP3. Interestingly, the IDHwt astrocytoma TP1 cases (*) we analyzed are classified as mesenchymal or classical, which are both the worst survival prognosis groups in the Verhaak classification^29^. This indicates the Verhaak classification agrees with both the mutation and TP subtypes and disagrees with histology in terms of survival prognosis for these cases. In contrast, Verhaak classifies the IDHmut-non-codel glioblastoma TP1 cases (†) as proneural, which is associated with better prognosis. In this case, Verhaak classification agrees with the mutation classification and disagrees with both the TP subtypes and histology, even though the survival prognosis for these cases favors the latter classification. Our transcriptomic classification is distinct from the method reported by Verhaak et al. Our method also reflects the mutation system and patient survival prognosis more closely than the Verhaak classification.

### ETC groups are similar to TCGA methylation subtypes

We compared our ETC classification to the six DNA methylation clusters described by TCGA^9^, LGm1 through LGm6. We refer to these six groups as the “TCGA methylation” classification. In the TCGA cohort, the concordance between our two methods was 87% while the concordance between the IDH-codel and the TCGA methylation classification was 94% (Fig. 2A, Supplementary Table 5). LGm1 cases were either classified as TP1 (15/48) or TP3 (29/48). LGm2 cases were mostly TP3 (162/228), with some classified as TP2a (43/228). LGm3 cases were almost all TP2a (107/115). Almost all LGm4 (137/138), LGm5 (228/232), and LGm6 (46/57) cases were TP1.

The IDHwt astrocytoma TP1 cases (*) we analyzed are classified as LGm4 or LGm5, which are both associated with worse survival prognosis in the TCGA methylation classification^29^. This indicates the TCGA methylation classification agrees with all other molecular classifications and disagrees with histology in terms of survival prognosis for these cases.

TCGA methylation classifies the IDHmut-non-codel glioblastoma TP1 cases (†) as LGm1, which is associated with worse prognosis. In this case, TCGA methylation classification agrees with both our TP subtypes and histology and disagrees with the mutation and Verhaak classifications. The former set of classifications better reflect survival prognosis. Our transcriptomic classification is similar to the TCGA methylation classification.

### ETC groups contribute additional survival information to previous molecular classifiers

**Figure 3:**
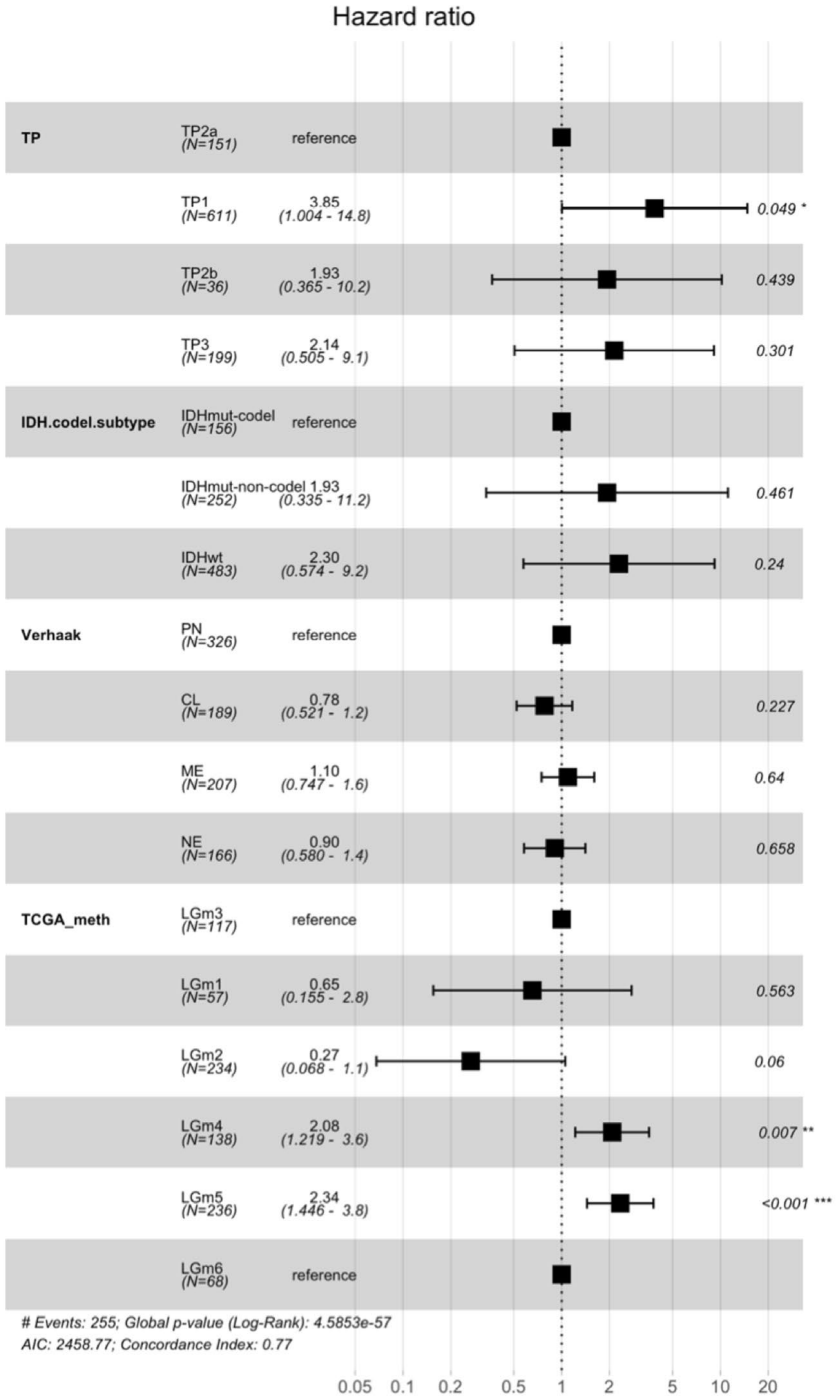
Forest plot of full cox regression model including transcriptome profile, IDH-codeletion subtype, Verhaak classification, and TCGA DNA methylation classification using TCGA data. For Verhaak, PN is proneural, CL is classical, ME is mesenchymal, and NE is neural. Hazard ratios are followed by the 95% confidence interval in paratheses. The last column shows p-values from the cox regression models, and * shows p < 0.05.

In order to discern the distinct contributions of each molecular classifier to survival prognosis, we developed a multivariable cox regression model which includes TP, mutation, Verhaak, and TCGA methylation subtypes. We found that only the TP1 group from our classification and the LGm4 and LGm5 groups from the TCGA methylation classification remained significant for survival prognosis in the full survival model (Fig. 3). Thus, our TP1 group provides additional survival information to the previous molecular classification methods. The combination of our TP groups and the TCGA methylation groups provides more survival information than mutation classification, the current standard.

### Molecular characteristics of the ETC groups

**Figure 4:**
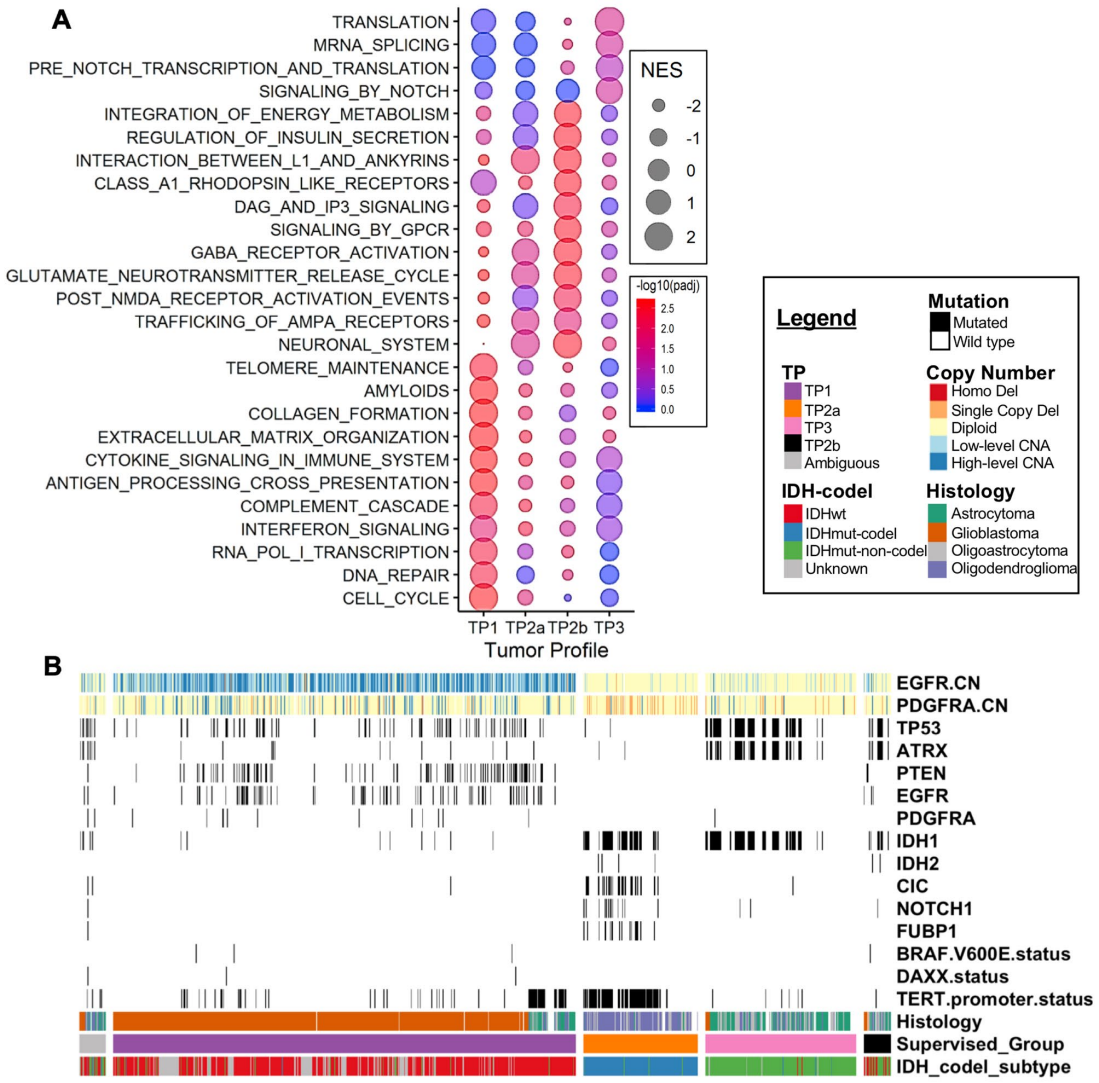
Molecular characteristics of TCGA Glioma TP subtypes. **(A)** GSEA significantly enriched pathways in each transcriptome profile visualized as a bubble chart, with normalized enrichment score (NES) as size of the bubble, and the negative log10 of BH-adjusted p-value as the shade of the bubble (blue low, red high). Significant pathways are those with BH-adjusted p values < 0.05. Pathways were manually selected and grouped for visual clarity. See complete list in Supplementary Table 5. **(B)** Oncoprint of significantly mutated genes for each transcriptome profile in the TCGA dataset. Chi-squared test with BH p-value adjustment was applied to the TCGA mutation data one vs rest for each group. Eight significantly mutated genes are shown.

Gene set enrichment analysis (GSEA) of TCGA transcriptomic data revealed TP1 is enriched for cell cycle, DNA repair, transcription, immune, ECM, and telomere maintenance pathways. TP2b is enriched for neurotransmission, GPCR signaling, and insulin secretion pathways. TP2a is enriched for similar pathways as TP2b albeit less significant. TP3 is enriched for immune, cell cycle, NOTCH signaling, transcription, and translation (Fig. 4A). Full GSEA pathway analysis is presented in Supplementary Table 6.

TP1 is enriched for TERT promoter, EGFR, PTEN, and TP53 mutations. All of these mutations are known to be associated with IDHwt glioblastomas^9,19^. TP2a is enriched for TERT promoter, FUBP1, NOTCH1, CIC, IDH1, and IDH2 mutations. *FUBP1* and *CIC* are both often mutated in TP2 and are known to be associated with oligodendroglioma^53^. *NOTCH1* mutation has been more recently associated with 1p/19q codeletion^8^ and may play a role in gliogenesis^54^ but its role in cancer development is unclear. TP3 is enriched for IDH1, ATRX, and TP53 mutations, all of which have reported associations with IDHmut-non-codel gliomas. DAXX, BRAFV600E, and PDGFRA mutations were not enriched in any groups. TP2a is enriched for PDGFRA copy number deletions (26%) and TP1 is enriched for EGFR copy number amplifications (88%) (Supplemental Table 7, Fig. 4B).

Amongst the IDHmut glioblastoma TP1 cases, 13 had available mutation data. Like other IDHmut-non-codel cases, this group is enriched for IDH1, ATRX, and TP53 cases. Like IDHwt cases, this group is enriched for TERT promoter mutations although at a lower proportion (25% vs 95%). Thus, the mutational profile of IDHmut TP1 cases is more similar to IDHmut/TP3 cases than to IDHwt/TP1 cases (Supplemental Table 7).

### Validation of supervised classifier on REMBRANDT

**Figure 5:**
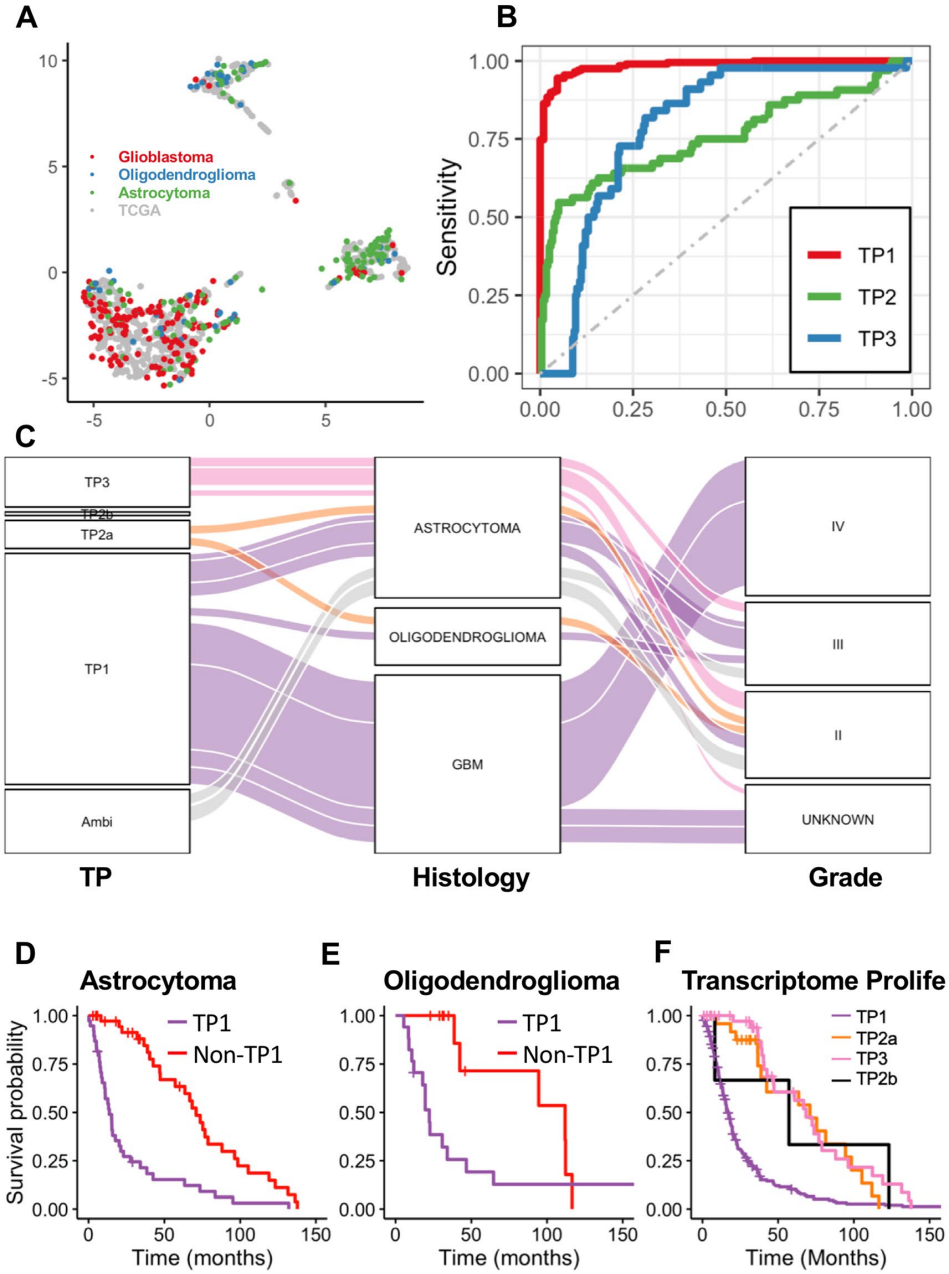
Validation of the supervised classifier using the REMBRANDT dataset. (**A**) UMAP embedding from standardized TCGA Glioma gene expression data with standardized REMBRANDT microarray projected on the embedding. The embedding used 161 genes which were present in both the REMBRANDT and TCGA gene expression datasets of the 168 selected for the ETC algorithm. Colors indicate histological diagnosis for REMBRANDT samples and TCGA samples are grey. (**B**) Receiver operating characteristic (ROC) curve for one vs rest analysis for TP1, TP2a, and TP3 comparing ETC predictions to DBU gold standard results for the REMBRANDT data. Area under ROC (AUROC) for TP1, TP2a, and TP3 are 0.99, 0.76, and 0.79, respectively. (**C**) Alluvial diagram showing the distribution, consistencies and discrepancies of REMBRANDT samples in each of four classifications, histology and supervised transcriptome classification. Colors represent the transcriptome profile classification of the samples. (**D**) KM curve of overall survival for 78 REMBRANDT astrocytoma cases divided in TP1 and non-TP1 (LRT p = 1.4e − 6). (**E**) KM curve of overall survival for 30 REMBRANDT oligodendroglioma cases divided into TP1 and non-TP1 (LRT p = 0.02). (**F)** KM curve for transcriptomic subtypes (Likelihood ratio test p = 7e − 14).

The REMBRANDT data is a microarray dataset consisting of 395 samples diagnosed as WHO grade 2, 3, or 4 gliomas. UMAP embedding of the combined REMBRANDT gene expression and TCGA data validated four distinct transcriptomic clusters (Fig. 5A). ETC was applied to this independent microarray dataset for validation. There were 244 TP1, 33 TP2a, 3 TP2b, 53 TP3, and 62 ambiguous samples. The classifier predictions were compared to the DBU groups of the REMBRANDT dataset and the AUROC for TP1 vs rest was 0.99, TP2a vs rest was 0.76, and TP3 vs rest was 0.79 (Fig. 5B). TP2b could not be assessed due to low sample count. The mean classification confidence of the ETC algorithm was 0.97, 0.90, 0.66, and 0.93, for groups TP1, TP2a, TP2b, and TP3, respectively. This highlights the robustness of the classifier across different gene expression platforms. Comparison of ETC to histology revealed that TP1 includes glioblastoma (141/200) and astrocytoma (42/200) cases, TP2a includes astrocytoma (12/24) and oligodendroglioma (11/24) cases, TP2b includes astrocytoma (3/3) cases, and TP3 mostly includes astrocytoma (34/43) cases (Fig. 5C, Supplementary Table 8).

We noted more astrocytoma and oligodendroglioma cases were classified as TP1 than expected and assessed if this reclassification from ETC reflected survival differences. TP1 cases that are histologically astrocytoma or oligodendroglioma have worse survival prognosis than non-TP1 cases (Fig. 5D,E). In fact, ETC is more predictive of survival than histological diagnosis and WHO grade (Chi-squared p < 2.2e − 16 for both).

## Discussion

We developed and validated an unbiased, automated pipeline for transcriptomic clustering. Without any domain knowledge, our classifier recapitulated known glioma subtypes from histology and mutation status. Our analytical pipeline avoids the potential of overfitting a supervised model to misclassified or mishandled samples^21,22^, and can be used in establishing gold standard datasets devoid of erroneous and questionable samples for the development of automated tumor classifiers (Fig. 1).

We developed supervised classifiers from TCGA data and validated with the REMBRANDT microarray dataset (Fig. 5). Most previous studies utilized one optimized classifier, which is prone to overfitting during training. To reduce classification dependency on specific genes or models, we advocate the use of many models, each with a small number of genes. This approach distributes the classification task across a large number of models and will democratize the contribution of genes to this task. Our classifier, ETC, is composed of 1000 models of 38 genes on average selected from the 168 genes, allowing for the assessment of classification confidence for each patient.

ETC clustered all TCGA samples except 17 into four transcriptome profile groups. Characteristics for TP1 cases include IDHwt, glioblastoma (Fig. 2A), high immune infiltration and cellular proliferation (Figs. 1E, 3A), and poor survival prognosis (Fig. 1F). An intriguing 27 TP1 gliomas were classified as IDHmut but histologically glioblastoma (Fig. 2A). The worse prognosis of this group compared to other IDHmut suggests these are secondary glioblastomas, or tumors originally IDHmut astrocytoma which recurred and developed into the higher-grade glioblastoma. The overall mutational profile of this subset reflects the IDHmut over a IDHwt except that these 27 TP1 cases are enriched for TERT promoter mutations which are nearly prevalent in IDHwt cases (Fig. 4B). This finding supports the important role of TERT promoter mutation in glioma progression. Their classification into TP1 provides additional prognostic value to IDH mutation status, although histology can already identify these cases as worse prognosis. While some of these TP1 IDH-mut glioblastoma cases are classified as LGm1 (worse prognosis), others are classified as LGm2 (better prognosis, Fig. 2A). This indicates methylation-based classification does not identify all potential secondary glioblastomas whereas both histology and transcriptomic methods may be more comprehensive.

Our study can explain the transcriptomic nature of the discrepant groups between histology and mutation. Specifically, both IDHmut glioblastomas and IDHwt astrocytomas are both TP1 and therefore transcriptionally more similar to grade IV glioblastomas.

TP2a is characterized as IDHmut-codel, oligodendrogliomas (Fig. 2A) with high tumor purity (Fig. 1D). Meanwhile, TP2b samples express more normal neuronal function genes, like voltage gate channels and AMPAR receptors (Fig. 4A), and they have significantly lower tumor purity without an associated increase in immune cell infiltration (Fig. 1E,F). This information suggests that TP2b tissue is mostly composed of neurons and few infiltrating malignant cells. TP3 exhibit increased NOTCH signaling (Fig. 4A), are astrocytoma and IDHmut-non-codel (Fig. 2A). A recent report showed IDH mutation causes decreased immune infiltration and anti-tumor immunity^55^. Our data agree with these findings, since the IDH mutation associated groups TP2a and TP3 have higher tumor purity and lower immune infiltration compared to the other groups in TCGA data. Interestingly, while TP2a was enriched for NOTCH1 mutations, TP3 was enriched for NOTCH signaling gene pathways (Fig. 4). The controversial role of NOTCH signaling in gliomagenesis has been noted in the literature^56^.

Our classification is unique from the transcriptomic classification reported by Verhaak et al.^29,35^ in that the “proneural” group includes tumors from TP1, TP2a, and TP3 while the “mesenchymal”, and “classical” groups mostly include TP1 tumors. However, it is similar to the DNA methylation classification reported by TCGA^9^. TP1 mostly includes LGm4, LGm5, and LGm6, TP2a mostly includes LGm3, and TP3 mostly includes LGm2 (Fig. 2A). The similar classification between these methods suggests that global DNA methylation and gene expression are correlated in glioma tumors and that regardless of the three GBM DNA methylation subtypes, they are similar at the gene expression level. Additionally, although DNA methylation is becoming widely adopted for CNS tumor classification, it is beneficial to have complimentary methods for classification especially since RNASeq equipment are more widely available compared to DNA methylation, so future clinicians and scientists can use either technique for initial classification.

The validity of ETC was demonstrated using gene expression data from a 395-case microarray dataset^36^. The classifier confidently assigns most samples to one of the TP groups, a remarkable observation given the difference in array platforms. More importantly, survival is consistent with patient’s TP status, even within histological subtypes, further validating the utility of ETC (Fig. 5).

In conclusion, transcriptomic profiling provides an unbiased classification method that can aid in tumor classification. When applied to glioma transcriptome data, the method agreed with the major classification methods, including the WHO integrated histology and mutation classification as well as methylation-based classification of gliomas.

## Supplementary information

Supplementary tables.
